# Weaver syndrome: A report of a rare genetic syndrome

**DOI:** 10.4103/0971-6866.50869

**Published:** 2009

**Authors:** Nitin Bansal, Amit Bansal

**Affiliations:** M.S. Orthopedics, Senior Resident, Department of Orthopedics, AIMSR, Bathinda; M.D. Pulmonary Medicine, Senior Resident, Department of Pulmonary Medicine, GMC, Chandigarh, India

Sir,

We studied a 29-year-old female patient admitted in our Gynecology and Obstetrics department. Ours was a case with craniofacial malformations, limb abnormalities, psychomotor retardation, and low pitched voice. Craniofacial malformations include a broad forehead, flat occiput, prominent eyes, glossoptosis, large low-set ears, hypertelorism, long philtrum, small round chin, and relative micrognathia. Limb abnormalities include prominent finger pads, camptodactyly, broad thumbs, thin and deep-set nails, clinodactyly, limited elbow and knee extension, wide distal long bones, and foot deformities (pes cavus and metatarsus adductus). Family history of the patient includes a sister with similar facial features who died at the age of 35 years. The remaining family history is negative.

Routine blood tests were normal. The karyotype was 46 XX. 5q35 -. X-rays of the skull, all long bones, foot, and hand confirm our clinical findings. Advanced bone age was clear in the wrist X-ray and metaphysis flare was present.

Weaver syndrome[1] was first described by Dr. David Weaver in 1974. Weaver syndrome is rare. About 30-50 cases have been published in the medical literature. It occurs in both males and females. A numbers of different symptoms occur in Weaver syndrome; however, it primarily results in rapid growth beginning in the prenatal period and continuing through the toddler years and into the elementary school years. It is a syndrome of overgrowth of prenatal onset, advanced bone age[3], retarded psychomotor development, widened distal long bones, camptodactyly, pes cavus[5] and distinctive craniofacial appearance marked by large ears, broad forehead, hypertelorism, and long philtrum. Other features included psychomotor delay, looseness of skin, pes cavus[5] and hernias. Weaver syndrome is considered a variant of the Marshall-Smith syndrome but, according to some authors, these are separate entities that share some common features, including abnormal bone maturation, accelerated growth, and delayed development, but differ in their craniofacial dysmorphism. Widened middle and proximal phalanges, failure to thrive, craniofacial abnormalities (small face, prominent eyes, blue sclera, flat nose with anteverted nares, choanal atresia, and glossoptosis), respiratory disorders, hypertrichosis, and early death uniquely characterize the Marshall-Smith syndrome. A syndrome marked by symptoms similar to those in the Weaver syndrome, with hyperprogesteronemia and maternal luteoma and one with cleft lip, accessory nipples, pectus excavatum, bifid xiphoid process, abnormal vertebral bodies, and inflexible right thumb are referred to as the Weaver-like syndrome. Weaver syndrome is, for the most part, a sporadic condition, meaning that a child affected by it did not inherit it from a parent. In a very few families, autosomal-dominant inheritance[7] has been reported, which means that both a parent and his/her child is affected by the Weaver syndrome. The cause of Weaver syndrome is not known and the gene(s) that is involved in it has not been identified. Quite recently, a number of children have been described with features of both Weaver syndrome and neurofibromatosis, raising the possibility of a contiguous gene syndrome[4] on chromosome 17 resulting in both conditions due to a deletion of a small piece of DNA containing the genes for both disorders. No molecular explanation for this cooccurrence has been found but should become clear in the next few years.

The nuclear receptor SET domain-containing protein 1 (NSD1) gene[2] encodes a histone methyltransferase and is located on chromosome 5q35. NSD1 was initially isolated in a search for proteins that interact with the ligand-binding domain of retinoic acid receptor α and subsequently was shown to be the fusion partner of NUP98 in some cases of childhood acute myeloid leukemia. NSD1 contains multiple functional domains, including SU(VAR)3–9, E(Z), trithorax (SET), and SET-associated (SAC) domains that together mediate the histone methyltransferase activity of NSD1. A C5HCH and five plant homeodomains, which are implicated in chromatin regulation and are zinc finger-like motifs characterized by cysteine and histidine residues, and two proline-tryptophan-tryptophan-proline (PWWP) domains, which may mediate protein-protein interactions, are often found in proteins that act at the chromatin level. NSD1 also contains two nuclear receptor interaction domains (NIDs), NID^−L^ and NID^+L^, which are typical of those found in nuclear receptor corepressors and coactivators, respectively. The functions of NSD1 are not known, but it has been shown to methylate both H4 K20 and H3 K36, modifications that are individually associated with transcriptional repression. The presence of two distinct NIDs has given rise to the hypothesis that differential ligand binding to NID^−L^ and NID^+L^ allows NSD1 to both negatively and positively regulate transcription. It has been proposed that *NSD1* may cause other overgrowth phenotypes, such as Weaver syndrome, Sotos syndrome[6], and BWS. We therefore believe that a diagnosis of Weaver syndrome should be given only if the presence of *NSD1* abnormalities has been excluded, otherwise it is labeled as Sotos syndrome.
